# Nutritional Support for Patients Sustaining Traumatic Brain Injury: A Systematic Review and Meta-Analysis of Prospective Studies

**DOI:** 10.1371/journal.pone.0058838

**Published:** 2013-03-19

**Authors:** Xiang Wang, Yan Dong, Xi Han, Xiang-Qian Qi, Cheng-Guang Huang, Li-Jun Hou

**Affiliations:** 1 Department of Neurosurgery, Changzheng Hospital, Second Military Medical University, Shanghai, China; 2 Neuroscience Center, Changzheng Hospital, Second Military Medical University, Shanghai, China; 3 Department of Neurosurgery, Huashan Hospital, Fudan University, Shanghai, China; University of Pittsburgh, United States of America

## Abstract

**Background:**

In traumatic brain injury (TBI), the appropriate timing and route of feeding, and the efficacy of immune-enhancing formulae have not been well established. We performed this meta-analysis aiming to compare the effects of different nutritional support modalities on clinical outcomes of TBI patients.

**Methods:**

We systematically searched Pubmed, Embase, and the Cochrane Library until October, 2012. All randomized controlled trials (RCTs) and non-randomized prospective studies (NPSs) that compared the effects of different routes, timings, or formulae of feeding on outcomes in TBI patients were selected. The primary outcomes included mortality and poor outcome. The secondary outcomes included the length of hospital stay, the length of ventilation days, and the rate of infectious or feeding-related complications.

**Findings:**

13 RCTs and 3 NPSs were included. The pooled data demonstrated that, compared with delayed feeding, early feeding was associated with a significant reduction in the rate of mortality (relative risk [RR] = 0.35; 95% CI, 0.24–0.50), poor outcome (RR = 0.70; 95% CI, 0.54–0.91), and infectious complications (RR = 0.77; 95% CI, 0.59–0.99). Compared with enteral nutrition, parenteral nutrition showed a slight trend of reduction in the rate of mortality (RR = 0.61; 95% CI, 0.34–1.09), poor outcome (RR = 0.73; 95% CI, 0.51–1.04), and infectious complications (RR = 0.89; 95% CI, 0.66–1.22), whereas without statistical significances. The immune-enhancing formula was associated with a significant reduction in infection rate compared with the standard formula (RR = 0.54; 95% CI, 0.35–0.82). Small-bowel feeding was found to be with a decreasing rate of pneumonia compared with nasogastric feeding (RR = 0.41; 95% CI, 0.22–0.76).

**Conclusion:**

After TBI, early initiation of nutrition is recommended. It appears that parenteral nutrition is superior to enteral nutrition in improving outcomes. Our results lend support to the use of small-bowel feeding and immune-enhancing formulae in reducing infectious complications.

## Introduction

Traumatic brain injury (TBI) remains a major worldwide health and socioeconomic problem. It is the most common cause of death and disability in people between 15 and 30 years of age [1]. The weighted average mortality for severe TBI is 39%, and for unfavorable outcome on the Glasgow Outcome Scale (GOS) is 60% [2].

TBI has a dynamic pathophysiology that evolves in time, consisting of primary injury, followed by a combination of systemic disorders (hypoxia, hypotension, and hypercarbia) and local events, which together lead to secondary injury [2]. As brain is the functional regulator for metabolic activities, a complex milieu of metabolic alterations may occur in TBI, consisting of hormonal changes, aberrant cellular metabolism, and inflammatory cascade [3]. The abnormal metabolic processes, mainly including hypermetabolism, hypercatabolism, and glucose intolerance, have been recognized as incredibly essential elements of secondary injuries [3]–[5]. Not only can they complicate the initial period of hospitalization and stabilization, but also they may negatively impact rehabilitative treatments [3]. Nutritional support, in addition to providing daily calories, has been appreciated as an important adjunctive therapy for metabolic disorders following TBI [6].

Nutritional support constitutes an important issue in intensive care for critically ill patients. However, it is generally neglected and underestimated in the subgroup of TBI population. In the recent most important trials in nutrition, Casaer et al. only included 0.6% of patients with neurological diseases [7]. Heidegger et al. incorporated 15% of neurological patients, and only included those with functioning gastrointestinal tract [8]. Interestingly, disagreement on the role of early parenteral nutrition even existed between the two trials. Currently, nutritional support for TBI patients, especially the appropriate timing, route, and formula of feeding, has not been well illustrated yet. In the earlier Cochrane Review, a trend towards better outcome with early nutritional support for TBI patients was shown, but without any statistically significant result [6]. In addition to not including updated studies, it had several flaws of its own. Although the Brain Trauma Foundation has recommended achieving full caloric replacement by day 7 following TBI, no agreement has been reached in the optimal timing or route of feeding [9]. In fact, nutritional support is frequently underestimated in the clinical management of severe TBI patients. Given insufficient previous evidence, as well as the introduction of recent randomized controlled trials (RCTs), we perform this meta-analysis and systematic review, aiming to compare the effects of different timings and routes of feeding, and to explore the effect of immune-enhancing formula on outcomes in TBI patients.

## Methods

### Search Strategy

The overview of this meta-analysis was conducted in accordance with the Preferred Reporting Items for Systematic Reviews and Meta-analysis (PRISMA) statement **(checklist S1**) [10]. A computerized bibliographic search for all relevant articles from 1980 to October 2012 was performed by Ovid Medline, EMBASE, and the Cochran database. We used the following search core terms: “traumatic brain injury”, “craniocerebral injury”, “head injury”, “head trauma”, “enteral nutrition”, “enteral tube feeding”, “parenteral nutrition”, “gastrointestinal intubation”, “nutritional support”, “prospective cohort”, and “randomized controlled trial”. The language was limited to English. We also manually searched the references of selective papers to identify additional potentially eligible studies.

### Selection Criteria

Studies were selected into the meta-analysis if they: i.) were RCTs or non-randomized prospective studies (NPSs); ii.) investigated TBI patients; iii.) compared the effect of different feeding routes (enteral nutrition [EN] vs. parenteral nutrition [PN], or nasogastric enteral feeding vs. non-nasogastric enteral feeding), different feeding timings (early or delayed), or different immunonutritional elements (such as probiotics, arginine, glutamine, nucleotides, and ω-3 fatty acids), on the outcome variables; iv.) reported the number of outcome events in different interventions.

### Data Extraction

Two assessors (XW and YD) independently reviewed the full manuscripts of eligible studies. Data were extracted in standardized data-collection forms. Extracted data included first author’s name; year of publication; sample size; patients’ characteristics (mean age, gender); starting time of feeding; treatment arms; outcome variables and the score of quality assessment. Any discrepancy was resolved by discussion or a third author (CGH). Selected RCTs were critically appraised using the Jadad scale, which scores studies’ description of randomization (2 points), blinding (2 points) and attrition information (1 point) [11]. The Newcastle–Ottawa scale (NOS) was used to evaluate the methodological quality of prospective cohort studies, as recommended by the Cochrane Non-Randomized Studies Methods Working Group [12]. The quality of a study was judged on the selection of the study groups, the comparability of the groups, and the ascertainment of the outcome of interest.

### Studied Outcomes

Primary outcomes of clinical importance included mortality and functional outcome on GOS score. Secondary outcomes include the length of stay (LOS) in hospital or ICU, and major complications. Infectious complications and feeding-related complications were assessed, respectively. We defined infectious complications as pneumonia (ventilator or non-ventilator-associated pneumonia and other lower respiratory tract infections), central nervous system (CNS) infection, bloodstream infection (laboratory-confirmed-bloodstream infections and clinical sepsis), or urinary tract infection. Feeding-related complications include feeding intolerance, aspiration, diarrhea, constipation and vomiting, and abdominal distention.

### Statistical Analysis

Data relating to outcomes were combined from pertinent studies. We used risk ratios (RR) and the associated 95% confidence intervals (CIs) to pool binary outcomes, including mortality, poor outcome and complications. Mean differences (MDs) with 95% CIs were used for continuous outcomes, which included the LOS in hospital or ICU, and the length of ventilator days. Review Manager 5.1.7 (Cochrane Collaboration, 2012) was used to process the meta-analysis. The Mantel-Haenszel method was used to test the significance of treatment effect, and the random-effects model was used to estimate the overall RRs. Heterogeneity of treatment effects between studies was statistically explored by the I^2^ statistic. I^2^ statistic of 0%–40% indicates unimportant heterogeneity, 30%–60% indicates moderate heterogeneity, 50%–90% indicates substantial heterogeneity, and 75%–100% indicates considerable heterogeneity [13]. Besides, we performed subgroup analyses based on the following factors which may contribute to the heterogeneity: study design, sample size, publication year, staring time of early nutrition, and different route of feeding. The sensitivity analyses were carried out by excluding studies one by one, or by employing a fixed effect model. All reported P values were two-sides, and P values less than 0.05 were deemed as statistically significant. The publication bias was examined visually by inspecting the funnel plots on Review Manager 5.1.7, and statistically by using the Egger’s regression model, calculated by Stata 12.0 (Stata Corporation, College Station, TX, USA).

## Results

**Figure 1:**
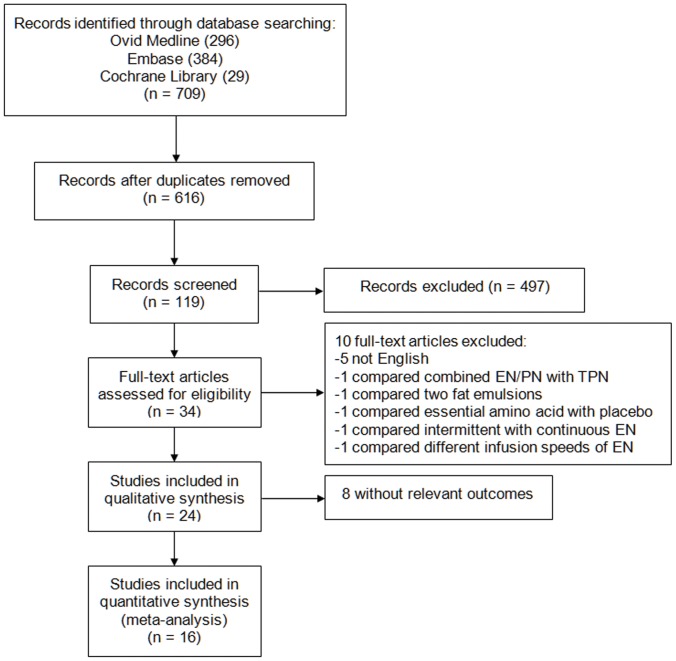
The flow diagram shows the selection of studies for the meta-analysis.

709 articles were found in total from the initial search, of which 34 eligible articles were selected after screening of titles and abstracts. Further, 10 studies were excluded, with 5 of non-English language, 1 comparing combined EN and PN with PN [14], 1 comparing two different fat emulsions [15], 1 comparing essential amino acid with placebo [16], 1 comparing intermittent EN with continuous EN [17], and 1 compared different infusion speed of EN [18]. In the remaining 24 studies included in qualitative synthesis, 8 articles lacked sufficient data relating to our outcomes [19]–[25]. Thus 16 studies were pooled into the meta-analysis, including 13 randomized controlled trials [26]–[38], and 3 NPSs [39]–[41]. The search flow diagram was shown in **Figure 1** **and protocol S1**. The characteristics of these studies were shown in **Table 1**. Of the 13 RCTs, the mean Jadad score was 2, ranging from 0 to 5. All NPSs have a high NOS score of 8.

**Table 1:** Characteristics of included studies of meta-analysis.

Author (year)	Sample size	Mean age, y	Male sex, %	Treatment arms (No.)	Timing from admission/injury to starting of nutrition	Initial GCS	Primary outcomes	Nutritional outcomes	Quality score#
Rapp (1983)	38	40	NA	Early PN (20) vs Delayed EN (18)	Early: within 48 hours after admission; delayed: till the bowel sounds were present and the gastric residual volume was less than 100 ml/hour	Mean: 7.5	Mortality; GOS	Nitrogen balance; nitrogen intake; caloric intake; nitrogen excretion; serum albumin level; serum transferrin values; lymphocyte count; serum glucose levels	R1B0A1 = 2
Hadley (1986)	45	28	88.9	Early PN (24) vs Early EN (21)	Within 48 hours of admission	≤10 (mean: 5.8)	Infection rate; GCS; mortality	Nitrogen loss; nitrogen intake; nitrogen balance; nitrogen excretion; serum albumin levels; weight loss	R0B0A1 = 1
Young (1987)	51	33	82.4	Early PN (23) vs Delayed EN (28)	Early: within 48 hours postinjury; delayed: till the termination of low wall suction	4–10	GOS; mortality; complication rate	Nitrogen balance; caloric balance; nitrogen intake; lymphocyte counts; albumin levels	R1B0A1 = 2
Grahm (1989)	32	27	90.6	Early jejunal (17) vs Delayed gastric (15)	Nasojejunal: within 36 hours of admission; gastric: after day 3 when gastric function returned	≤10	Infection rate; length of ICU stay	Nitrogen balance; caloric intake; nitrogen intake	R0B0A0 = 0
Borzotta (1994)	49	27	81.6	Early PN (21) vs Early EN (28)	Within 72 hours of injury	3–8	Complication rate	Measured resting energy expenditure; nitrogen balance; nutrition excretion	R1BOA0 = 1
Minard (2000)	27	33	70.4	Early EN (12) vs Delayed EN (15)	Early: within 60 hours of injury; delayed: when the gastroparesis resolved	3–11	Mortality; LOS in ICU and hospital; ventilator days; complication rate	Caloric intake	R1B0A1 = 2
Falcan (2004)	20	27	95	Early EN (10) vs Early EN with glutamine and probiotics (10)	Within 48 hours of admission	Mean: 7	Infection rate; LOS in ICU; period of mechanical ventilation	Nitrogen balance; caloric intake; protein intake	R1B0A1 = 2
Kostadima (2005)	41	47	78	Gastrostomy (20) vs Nasogastric tube (21)	Within 24 hours of intubation	<6	Incidence of VAP; length of stay in ICU; duration of mechanical ventilation; mortality	NA	R1B0A1 = 2
Briassoulis (2006)	40	10	NA	Immune enhancing diet (20) vs Regular formula (20)	Within 12 hours of admission	Mean: 6.2	Rate of nosocomial infections; LOS; length of mechanical ventilation; mortality	Cytokines concentrations; nitrogen balance	R2B1A1 = 4
Hartl (2008)	797	≥16	NA	Early nutrition (755) vs Delayed nutrition (42)	Early: within 7 days postinjury; delayed: after 7 days postinjury	3–8	Mortality	NA	S4C202 = 8
Khorana (2009)	40	41	80	Standard EN (20) vs Immunonutrient containing EN (20)	Within the first 24 hour after operation	5–10	Complication rate; LOS in ICU	IL-6 and IL-10 levels	R2B2A1 = 5
Acosta-Escribano (2010)	104	38	86	Transpyloric feeding (50) vs Gastric feeding (54)	Within the first 24 hour after admission	Mean: 6	Incidence of VAP; GI complications; length of stay in ICU and hospital; mortality	Efficacious volume of diet	R1B0A1 = 2
Justo Meirelles (2011)	22	31	90.9	Early PN (10) vs Early EN (12)	Early after admission when hemodynamically stable	9–12	Mortality; complication rate; LOS in ICU	Calories intake; nitrogen intake; nitrogen balance; urinary nitrogen loss; serum glucose; CRP; albumin	R1B0A1 = 2
Chourdakis (2011)†	59	35	79.7	Early EN (34) vs Delayed EN (25)	Early: Within 24–48 hours after injury; delayed: when the gastroparesis resolved (48 h-5d)	≥9	Mortality rate; complication rate; LOS in ICU	TSH, FT3, FT4, cortisol, and testosterone levels	R1B0A1 = 2
Chiang (2012)†	297	0–99	72.4	EN (145) vs Non-EN (152)	Early: within 48 hours postinjury; delayed: no feeding formula added for 7 days	4–8	GOS; mortality; LOS in ICU	NA	S4C202 = 8
Dhandapani (2012)†	95	35	NA	Early EN (64) vs Delayed EN (31)	Early: within 7 days postinjury; Delayed: after 7 days postinjury	4–8	GOS	Anthropometric measurements (mid-arm circumference; triceps skin fold thickness); serum albumin levels; urinary creatinine levels; incidence of malnutrition	S4C2O2 = 8

† Non-randomized prospective studies.

# Jadad score for RCTs: randomization (R0-2), blinding (B0-2) and attrition information (A0-1); Newcastle-Ottawa Scale (NOS) for cohort studies: selection (S0-4), comparability (C0-2), outcome (O0-3).

Abbreviations: EN, enteral nutrition; GCS, Glasgow Coma Scale; GOS, Glasgow Outcome Score; LOS, length of stay; NA, not available; NNG, non-nasogastric feeding; NG, nasogastric feeding; PN, parenteral nutrition; vs, versus; VAP, ventilator-associated pneumonia.

### Early VS Delayed

**Figure 2:**
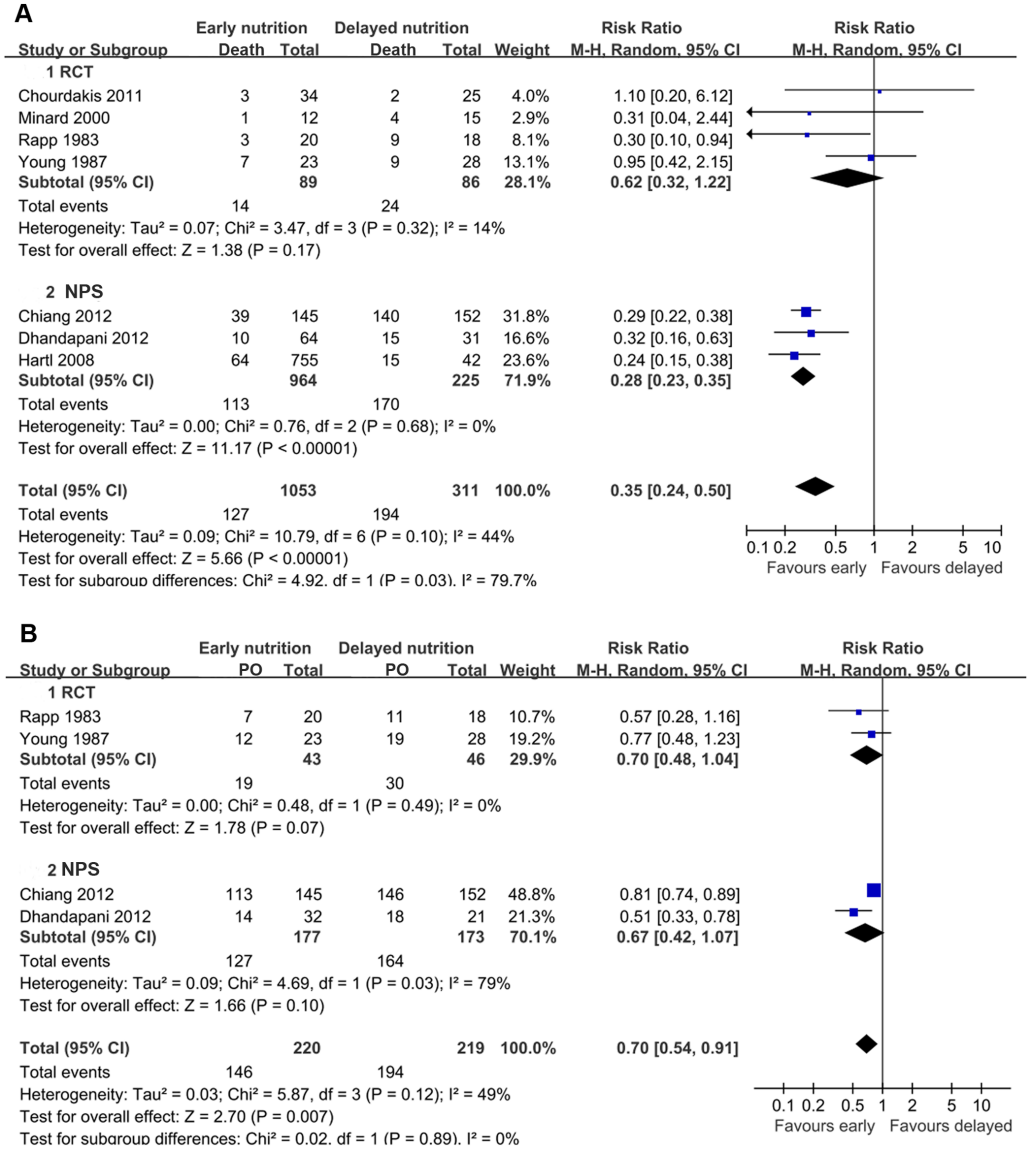
Comparison of the effect of early feeding and delayed feeding on outcomes in patients with TBI. (A) Forest plot illustrates the different effects on mortality. (B) Forest plot shows the different effects on poor outcome. PO, poor outcome.

8 studies were available for the comparison of early nutrition with delayed nutrition, including 5 RCTs [25], [26], [29], [31], [37], and 3 NPSs [39]–[41]. Research conducted by Grahm et al. compared early jejunal feeding with delayed gastric feeding without data of mortality or functional outcome, and thus was excluded from the pooled analyses [29]. The pooling data of the other seven studies indicated that early nutrition was associated with a significant reduction of the mortality rate compared with delayed nutrition, but with moderate heterogeneity (RR = 0.35; 95% CI, 0.24–0.50; P<0.05; I^2^ = 44%) (**Figure 2A**).

The heterogeneity was explored by subgroup analyses according to the sample size, publication year, study design, starting time of early feeding, and the route of feeding. Most studies have a small sample size below 100 except for two studies (**Table 1**). Five studies initiated the early nutrition within 72 hours after injury [26], [28], [31], [37], [40]. In two NPSs, mortality was compared in patients who started feeding within/out of the first 7 days postinjury. Three studies compared the early enteral nutrition with delayed enteral nutrition [31], [37], [41]. Two trials compared early PN with delayed EN [26], [28]. The results of subgroup analyses were shown in **Table 2**. Notably, the RCT subgroup, the subgroup of publication year earlier than 2005, and the subgroup that compared early PN with delayed EN were not found to be with statistical significances (RR = 0.62 with 95% CI 0.32–1.22, RR = 0.54 with 95% CI 0.23–1.27, and RR = 0.57 with 95% CI 0.19–1.76, respectively), demonstrating that these subset factors might be the sources of heterogeneity. Meta-regression was not further performed due to the limited number of available studies.

**Table 2:** Subgroup analyses for studies evaluating the effects of early nutrition and delayed nutrition on mortality.

Subgroups	N	RR (95% CI)	Heterogeneity (I^2^)
**Total**	7	0.35 (0.24, 0.50)	44%
**Sample size**			
<100	5	0.49 (0.27, 0.87)	30%
>100	2	0.28 (0.22, 0.35)	0
**Publication year**			
<2005	3	0.54 (0.23, 1.27)	35%
>2005	4	0.29 (0.23, 0.37)	6%
**Study design**			
RCT	4	0.62 (0.32, 1.22)	14%
NPS	3	0.28 (0.23, 0.35)	0
**Starting time of early nutrition**			
<72 h	5	0.45 (0.24, 0.87)	56%
<7 d	2	0.26 (0.18, 0.39)	0
**Compared route**			
Early PN vs. delayed EN	2	0.57 (0.19, 1.76)	62%
Early EN vs. delayed EN	3	0.37 (0.21, 0.68)	0
Early EN vs. no feeding	1	0.29 (0.22, 0.38)	-
Unknown	1	0.24 (0.15, 0.38)	-

Abbreviations: EN, enteral nutrition; N, number of studies; NPS, non-randomized prospective study; PN, parenteral nutrition; RCT, randomized controlled trial; RR, relative risk.

**Figure 3:**
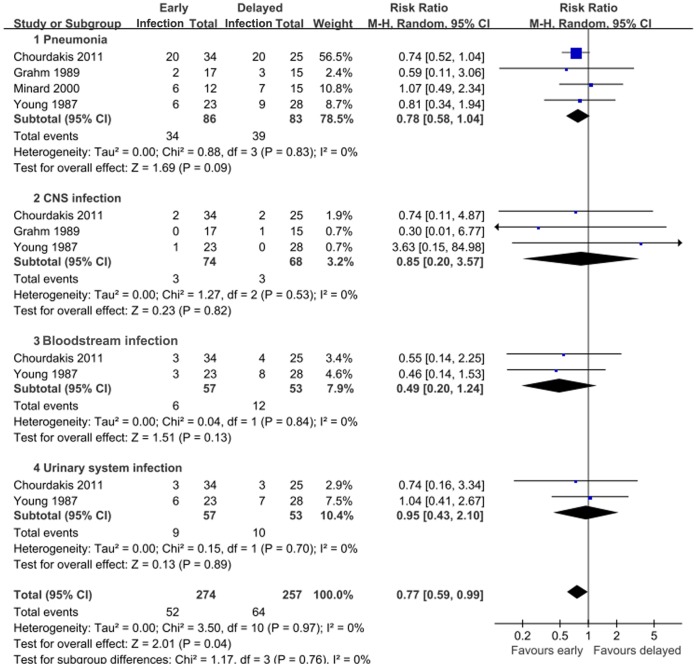
Comparison of the effect of early feeding and delayed feeding on infectious complications in patients with TBI.

Four studies reported the functional outcome on GOS. There was a significant lower risk of poor outcome in patients who received early nutrition compared with those who received delayed nutrition (RR = 0.70; 95% CI, 0.54–0.91; P<0.05) (**Figure 2B**). Four studies investigated the occurrence of infectious complications in both groups, in terms of pneumonia, CNS infection, bloodstream infection, and urinary tract infection. Generally, early nutrition was significantly associated with a lower risk of infectious complications compared with delayed nutrition (RR = 0.77; 95% CI, 0.59–0.99; P<0.05). In the subgroup analyses, however, no similar statistical significance was observed (**Figure 3**). Three studies reported the rate of feeding complications, including diarrhea and feeding intolerance. However, no statistical significance was revealed (**figure S1**). Two studies reported the mean value and standard deviation (SD) of length of stay (LOS) in intensive care unit (ICU) [31], [41], whereas no significant difference was observed between early and delayed groups (P = 0.68) (**figure S2**).

**Figure 4:**
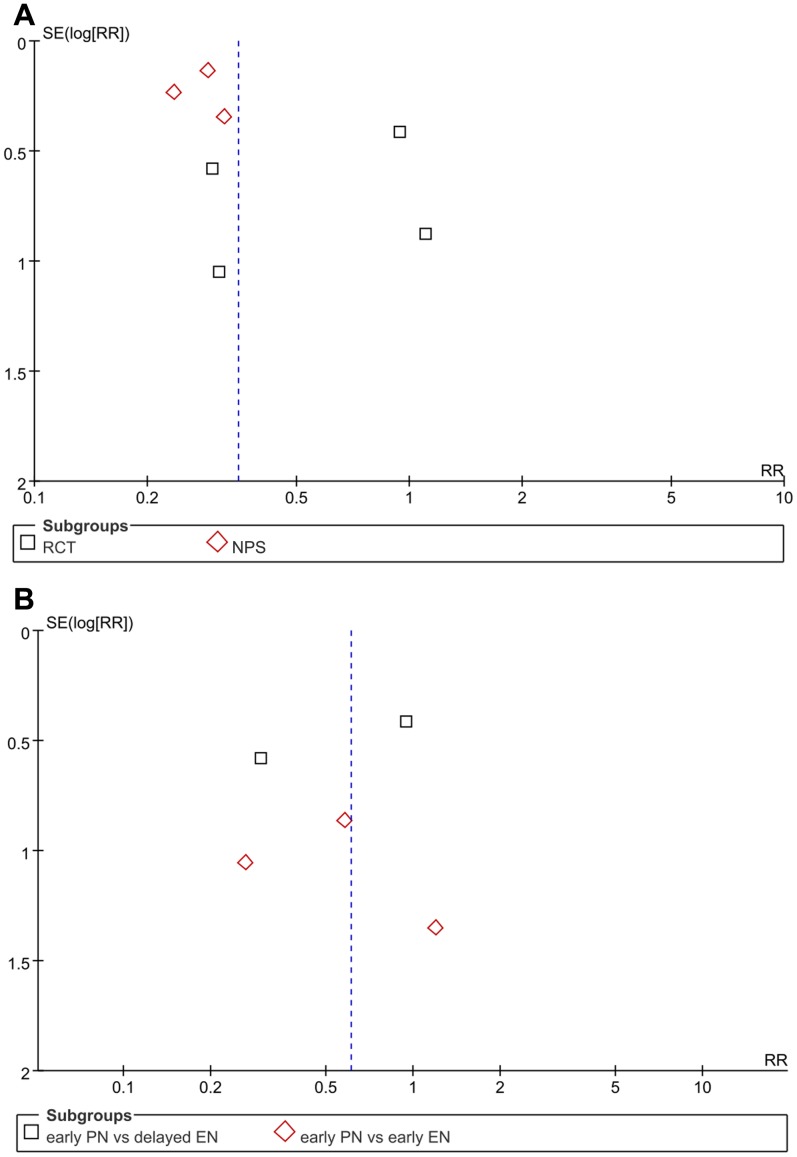
Funnel plots for the detection of publication bias. (A) Funnel plot of studies evaluating the effects of feeding timings on mortality, which is approximately symmetric. (B) Funnel plot of studies evaluating the effects of feeding routes on mortality, which appears to be symmetric.

The funnel plots of data relating to mortality were found to be symmetrical, suggesting a low likelihood of having publication bias (**Figure 4A**). No publication bias was revealed by Egger test either (P = 0.239).

### EN VS PN

Five RCTs involving a total of 215 patients evaluated delivery route of nutrition support (EN vs PN) in TBI patients (**Table 1**). As it showed before, two trials compared early PN with delayed EN [25], [26]. In the other three trials, both PN and EN were started early after admission [27], [30], [38].

**Figure 5:**
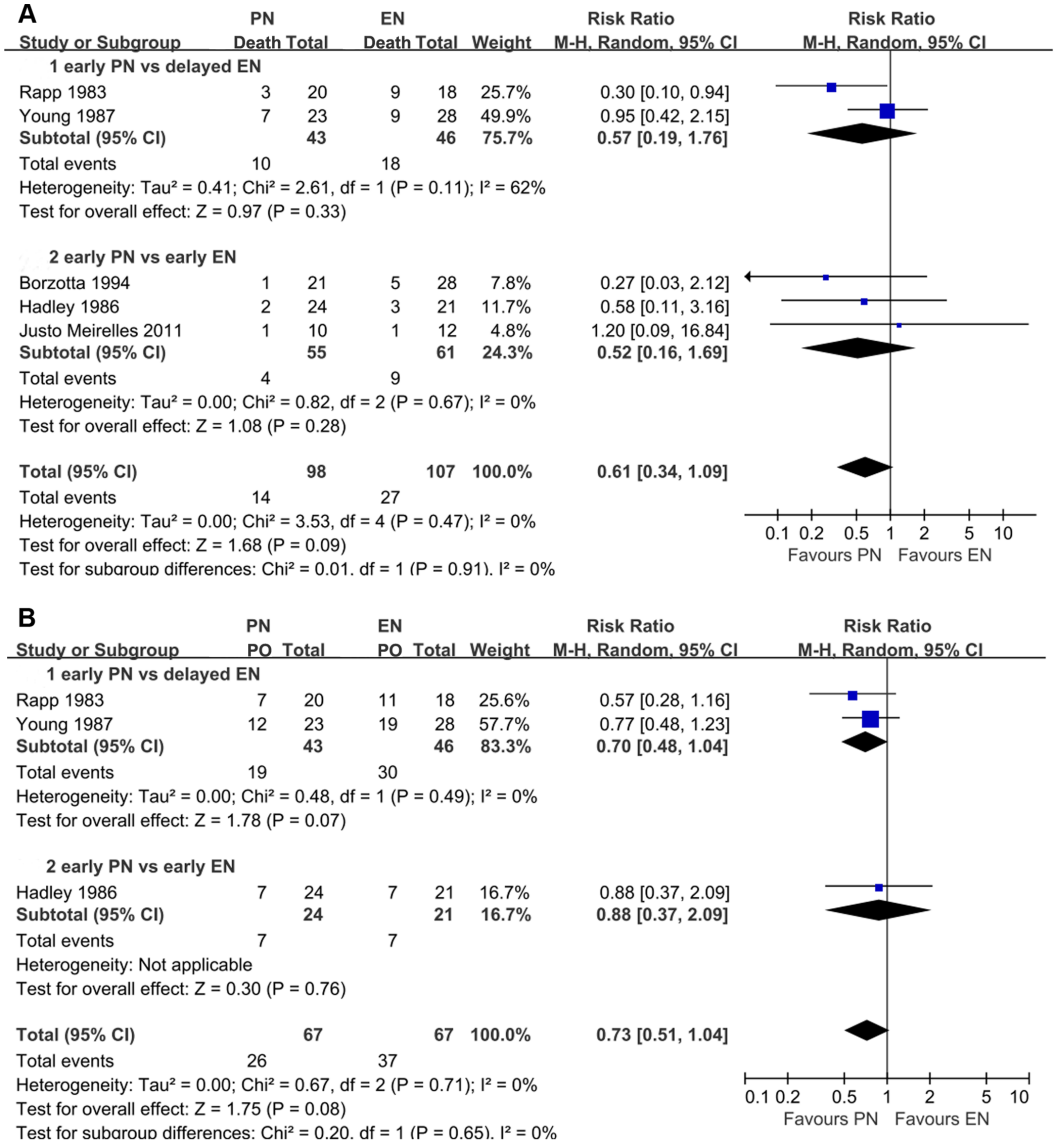
Comparison of the effect of enteral feeding and parenteral feeding on outcomes in patients with TBI. (A) Forest plot illustrates the different effect on mortality. (B) Forest plot shows the different effect on poor outcome. PO, poor outcome.

Aggregating data of the five studies demonstrated a trend toward a lower mortality rate associated with PN (RR = 0.61; 95% CI, 0.34–1.09; I^2^ = 8). Nevertheless, statistical significance was not revealed (P = 0.09) (**Figure 5A**). In order to investigate the impact of different starting time on the results, the subgroup analysis was performed. However, no statistically significant result was revealed in any subgroup (P>0.05) (**Table 3**). It is similar when subanalyzing the impact of publication year (**Table 3**). The low heterogeneity may justify the application of a fixed-effect model. In sensitivity analyses by using this model, a marginal statistical significance was revealed (RR = 0.56; 95% CI, 0.32–0.99; P = 0.05). Further, only by excluding the study by Young et al. [28], a statistical significance without heterogeneity was demonstrated (RR = 0.35; 95% CI 0.15–0.83; P = 0.02; I^2^ = 0).

**Table 3:** Subgroup analyses for studies evaluating the effects of parenteral nutrition and enteral nutrition on mortality.

Subgroups	N	RR (95% CI)	Heterogeneity (I^2^)
**Total**	5	0.61 (0.34, 1.09)	0
**Publication year**			
<1990	3	0.60 (0.29, 1.27)	24%
>1990	2	0.47 (0.09, 2.41)	0
**Compared timing**			
Early PN vs. delayed EN	2	0.57 (0.19, 1.76)	62%
Early PN vs. early EN	3	0.52 (0.16, 1.69)	0

Abbreviations: EN, enteral nutrition; N, number of studies; PN, parenteral nutrition; RR, relative risk.

**Figure 6:**
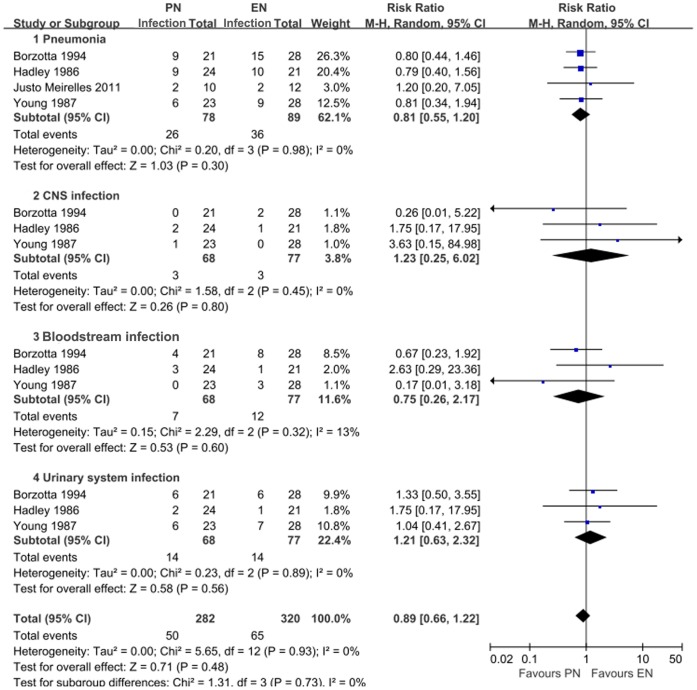
Comparison of the effect of enteral feeding and parenteral feeding on infectious complications in patients with TBI.

Three of the five studies reported the functional outcome on GOS. Pooling data revealed a trend toward reducing the rate of poor outcome in PN groups (RR = 0.73; 95% CI, 0.51–1.04). Nevertheless, statistical significance was not observed in the overall analysis or in any subgroup analysis according to the timing of nutrition (P>0.05) (**Figure 5B**). The fixed effect model also failed to show any significant alteration. The reported infectious complications mainly included pneumonia, central nervous system (CNS) infection, bloodstream infection, and urinary tract infection. Pooling data suggested that PN patients may have a slight trend of lower rate of infection complications compared with EN patients (RR 0.89; 95% CI, 0.66–1.22), especially in reducing the occurrence of pneumonia, whereas statistical significance was not revealed (P = 0.48) (**Figure 6**). The heterogeneity was at an unimportant level across our pooled analyses.

The funnel plots of data relating to mortality were found to be symmetrical, suggesting a low likelihood of having publication bias (**Figure 4B**). No publication bias was revealed by Egger test either (P = 0.621).

### Standard Formula VS Immune-enhancing Formula

Three trials compared the immune-enhancing formula (arginine, glutamine, probiotics, and ω-3 fatty acids et al.) with the standard formula of EN in TBI patients [32], [34], [35]. Infection rate was reported in all trials, and the aggregating data revealed that immune-modulating formula was associated with a statistical significant reduction in infection rate in contrast with the standard formula (RR = 0.54; 95% CI, 0.35–0.82; P<0.05) (**figure S3**).

### Non-nasogastric (NNG) Feeding VS NG Feeding

Five trials compared NNG feeding with NG feeding in EN support. Taylor et al. compared a mixed group (intestinal or gastric) with a standard gastric group, and thus was excluded [18]. Minard et al. compared early nasoenteric feeding with delayed NG feeding [31]. Grahm et al. compared nasojejunal feeding with NG feeding [29]. Kostadima et al. compared percutaneous endoscopic gastrostomy with standard EN in the occurrence of ventilator-associated pneumonia [33]. Escribano et al. randomized patients to receive transpyloric feeding or gastric feeding [36]. The pooling data of 4 trials showed a significant reduction in the occurrence of pneumonia in patients receiving NNG feeding compared with those receiving NG feeding (RR = 0.62; 95% CI, 0.40–0.96; P = 0.03). The NNG group was associated with a trend to reduce mortality rate and shorten ventilator day, whereas failing to show statistical significances. Three trials additionally reported the LOS in ICU, and the aggregated results revealed no statistical significance in the two arms (**figure S4**).

## Discussion

We carried out a comprehensive literature search to detect prospective studies, including RCTs and NPSs, to compare the effects of different routes, timings and formulae of nutritional support on clinical outcomes in TBI patients. Explicit criteria were utilized for study selection and methodological quality assessment.

In comparison of different timing for nutritional support, our meta-analysis demonstrated beneficial effects of early nutrition on reducing mortality, improving functional outcome, and decreasing infectious complications. Although a trend was indicated for early nutrition in lowering the risk of stratified specific infections, no statistical significance was revealed. There was also no significant difference in feeding-related complications between the two arms. Notably, opposite to pooled data of NPSs, the data of RCTs failed to demonstrate statistical significance in mortality. The RCTs were largely carried out in early years with smaller sample sizes and limited nutritional support approaches, which might contribute to this discrepancy. Similar concern may also explain the heterogeneity from publication year. The earlier Cochrane review also suggested a trend toward using early nutrition to reduce mortality and improve functional outcome by analyzing fewer studies, whereas without statistical significance [42]. Furthermore, several queries were concerned. For study by Hadley et al., the PN and EN were both started within 48 hours after admission, and thus it may not be reliable for considering it as the comparison of early nutrition with delayed nutrition [27], [42]. Additionally, they included one study that compared different infusion speeds of EN [18]. In light of the simultaneous initiation of EN in both groups, it is not likely to be justified to consider this trial as the comparison of nutritional timings, and thus we excluded it. The Brain Trauma Foundation has cautiously recommended achieving full caloric replacement by day 7 following TBI based on limited evidence [9]. Taken together, our results reinforced the inclination of early nutrition for TBI patients.

When compared with EN, our results showed that there might be a trend toward using PN to reduce mortality and improve functional outcome, whereas statistical significance was only marginally revealed when the fixed effect model was used. In the subgroup analyses based on timing of feeding and publication year, no statistical significant findings were revealed. Moreover, significant difference in the rate of complications was not recognized in any subgroup analysis between the two arms. In fact, EN and PN show unique advantages, respectively. The use of EN is superior to PN in patients with functioning gastrointestinal tracts [43]. Compared with PN, EN formulae may conveniently make use of more effective substrates to support cell and organ function, have a lower risk of hyperglycemia or hyperosmolarity, be administered at lower rates to avoid overfeeding, and better support the gut mass and barrier function. However, the use of enteral feeding in patients with gastrointestinal intolerance is associated with underfeeding and consequent malnutrition [43],[44]. Less than 70% of patients receive an adequate enteral caloric intake even in the most experienced and motivated ICUs [45]. In comparison, PN patients have benefits in obtaining more dependable nutrient bioavailability, getting nutrition effects in a shorter period, requiring no functional GT tract, and staying away from satiety, abdominal distention or other enteral feeding complications [43]. However, overfeeding (the administration of excess dextrose, fat, or calories) and refeeding syndrome (rapid feeding of patients with preexisting malnutrition) may occur, and thus induce a variety of metabolic complications, including hyperglycemia, hypertriglyceridemia, thiamine deficiency, hypervolemic, and hypercapnia [44]. In our pooled studies, enrolled patients unanimously experienced moderate or severe TBI, mostly with GCS lower than eight [25]–[27], [30], [38]. They are always comatose, intubated or mechanically ventilated, with malfunctioning parasympathetic and sympathetic system, disturbed hypothalamic-pituitary axis, elevated intracranial pressure (ICP), increasing endogenous opioids and endorphins, and widespread prescription of narcotics. All of these unfavorable factors may contribute to impaired GT function, delayed gastric emptying, and increased risk of EN intolerance [3]. In this context, PN might be superior to EN for initial life saving nutritional support. Notably, PN was unanimously initiated early after admission in related studies. Thus, our result should not be misunderstood as opposition to the suggestion that EN is preferable whenever possible with functional gastrointestinal tracts [46]. However, data of feeding related complications, especially the data of hyperglycemia were insufficient across included studies, and further persuasive evidence is warranted.

Given the prevalence of inconveniences for routine nasogastric EN, other alternative EN routes have been attempted, including nasojejunal feeding, percutaneous endoscopic gastrotomy feeding, and transpyloric feeding [29], [33], [36]. Pneumonia rate was shown to be significantly reduced by NNG, which may be associated with the prevention of aspiration by NNG feeding. There was also a trend toward reducing mortality rate and decreasing ventilator days, but failed to demonstrate statistical significances. It has been evaluated that at least 20% of TBI patients don’t tolerate enteral alimentation at all in the first week [29]. By using NNG route, patients will probably tolerate enteral feeding as well as avoid the hyperalimentation brought about by PN. Although data on feeding complications were scant, in light of its well tolerance and prevention of pneumonia, we are inclined to side with ESPEN guidelines, which suggested that when jejunal feeding can be carried out easily, it should be given [47].

Furthermore, our results showed that immune-enhanced formulae were associated with a significant reduction in infectious complications compared with standard formulae. Although a growing number of studies emphasized the importance of nutrition content of foods on post-injury recovery, studies relating to TBI are scant, especially according to our criteria of study design. In fact, the effect of immune-enhanced formulae has been widely investigated in general population, but with confusing and undefined conclusions for critically ill patients [48]. The ESPEN guideline has recommended that immune-modulating formulae, enriched with arginine, nucleotides, and ω-3 fatty acids, are superior to standard enteral formulae in trauma patients [47]. In contrast, the updated guidelines from the Canadian Practice Group in 2009 and the American Dietetic Association evidence analysis library did not recommend the routine use of immune-modulating diets in critically ill patients [48]. The most prominent controversy was the effect of immune-modulating diet on mortality and functional outcomes. For example, the use of arginine-contained formulae has shown greater mortality, and it is hypothesized that arginine may be converted to nitric oxide and thus contributed to hemodynamic instability [4]. Our results could only suggest the benefits of immune-modulating diet for TBI patients in reducing infectious complications. The effects on mortality or functional outcome could not be elucidated by the few studies, and further studies are warranted to investigate the effects of particular diets on the outcome of TBI.

We are aware of the limitations of this meta-analysis. Trials with statistically significant results may be more likely to be published and cited, and are preferentially published in English language journals [49]. We included only studies written in English language and therefore, may have missed relevant studies published in non-English language journals. Besides, several studies (e.g. Rapp et al. and Young et al.) are included in 2 or more of the reported analyses; this has a potential risk of overemphasizing positive results. Although results from Egger’s tests and funnel plots did not show evidence of publication bias, their capacities to detect bias was limited by the small number of studies [49]. The studies included were of relatively poor quality, with most of RCTs having a Jadad score of <3 (13/15). Only a few trials described the method of randomization [34], [35]. Blinded design was only described in two trials on immune-enhanced formulae [34], [35]. Although it seems difficult to conceal the route of nutritional support, studies with inadequate or unclear concealment of allocation may overestimate the intervention effect. The sample sizes were relatively small across included studies, especially in the RCTs. Small sample size might contribute to the failure of randomization and imbalance between clinical variables, and thus failed to detect the statistically significant effects. In fact, the compared different arms were not well-controlled. For example, the comparison of route for nutritional support would be more convinced if started simultaneously. However, it is commonly seen that EN was initiated late until the recovery of gastroparesis [26], [27]. In fact, in subgroup analyses of timing of feeding, two studies that compared early PN with late EN were both revealed to be the potential source of heterogeneity. Question may be raised that whether the effects found were related to the parenteral nutrition or perhaps more due to the early onset of nutrition versusing delayed onset. The impossibility to differentiate here was a substantial confounding factor in the interpretation of results. In the sensitivity analyses of route of feeding, the exclusion of study by Young et al. has led to a significant change of results. Notably, only this study initiated the EN support until the termination of low wall suction, which was non-conventionally performed and might contribute to the heterogeneity [28]. Furthermore, our meta-analysis was absent of aggregating various nutrition indexes, such as caloric intake, nitrogen intake, and nitrogen balance. In previous systematic reviews, it has been revealed that the measurement methods, definitions of metabolic abnormalities, and energy expenditure following TBI varied greatly, which may restrict the incorporation [50], [51]. Additionally, the included studies utilized different criteria for inclusion and exclusion. Especially, the differences in severity of disease across studies may explain some of the heterogeneity. For example, a number of studies specifically investigated patients with mechanical ventilation [32]–[34], [36]–[38]. All of these limitations restrict the strength of conclusions drawn from our meta-analysis.

Though disputes of optimal nutritional support would continue, we postulate that the optimal clinical decision in nutritional support should be personalized, in terms of the individual profile, including nutritional status, severity, complications, feeding tolerance, and day-to-day changes in clinical conditions. Last but not least, greater multidisciplinary efforts from nutritionists and clinicians are required for better management of the nutritional support for TBI patients.

### Conclusions

Our meta-analysis lends support to early initiation of nutritional support for TBI patients, which can decrease mortality, reduce complications and facilitate recovery. PN appears to be superior to EN in reducing mortality and improving outcome in the acute gut-intolerant phase of TBI. Immune-modulating formulae seem to be superior to standard formulae in reducing infectious complications. Small-bowel feeding was recommended if possible. However, our results should be interpreted with caution given the various limitations. Further well-designed RCTs are expected to clarify the optimal nutritional strategies for TBI patients.

## Supporting Information

Figure S1 **Forest plot shows the effect of early nutrition and delayed nutrition on feeding compliations.** (A) Forest plot illustrates the effect on diarrhea. (B) Forest plot illustrates the effect on feeding intolerance. I, intolerance.
(TIF)

Figure S2 **Forest plot shows the effect of early nutrition and delayed nutrition on length of stay in the intensive care unit.**
(TIF)

Figure S3 **Forest plot shows the effect of standard and immuno-modulated nutritional formulae on infectious complications.**
(TIF)

Figure S4 **Forest plot shows the effect of non-nasogastric and nasogastric enteral feeding on outcomes in patients with TBI.** (A) Forest plot illustrates the effect on pneumonia. (B) Forest plot shows the effect on mortality. (C) Forest plot shows the effect on ventilator days. (D) Forest plot shows the effect on length of stay in the intensive care unit. Pneumo, pneumonia.
(TIF)

Checklist S1 **PRISMA Checklist.**
(DOC)

Protocol S1 **PRISMA Flowchart.**
(DOC)
