# Supplementary figures and images for: Nutritional Support for Patients Sustaining Traumatic Brain Injury: A Systematic Review and Meta-Analysis of Prospective Studies
Source: PLoS One. 2013 Mar 19;8(3):e58838. doi: 10.1371/journal.pone.0058838 (PMC3602547; doi:10.1371/journal.pone.0058838)

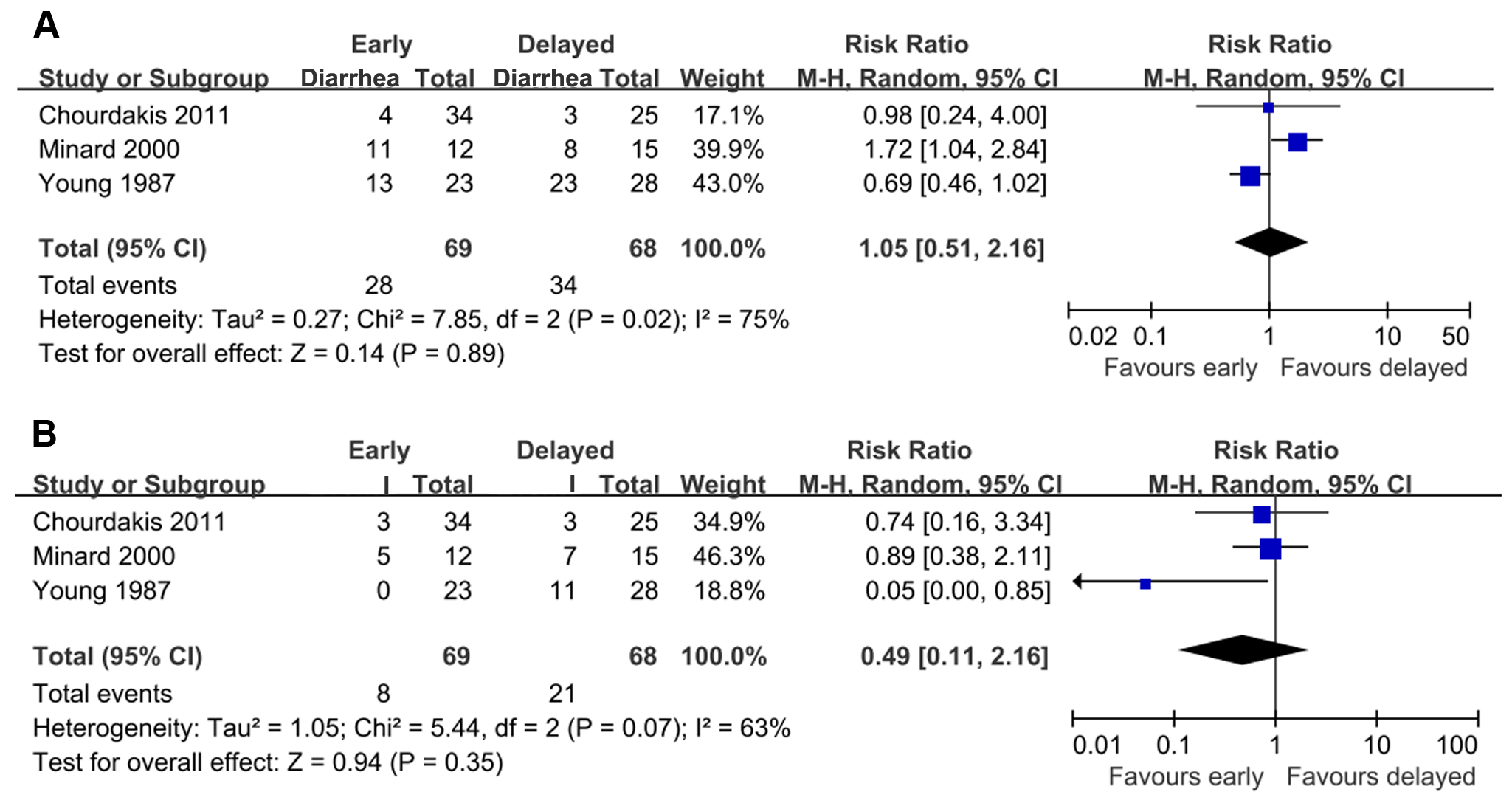

Supplement: Figure S1 — Forest plot shows the effect of early nutrition and delayed nutrition on feeding compliations. (A) Forest plot illustrates the effect on diarrhea. (B) Forest plot illustrates the effect on feeding intolerance. I, intolerance. (TIF) [file pone.0058838.s001.tif]

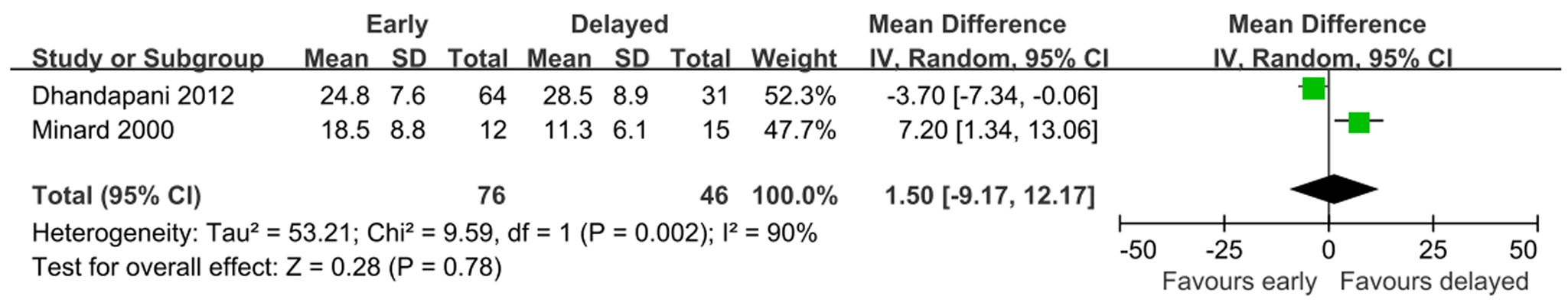

Supplement: Figure S2 — Forest plot shows the effect of early nutrition and delayed nutrition on length of stay in the intensive care unit. (TIF) [file pone.0058838.s002.tif]

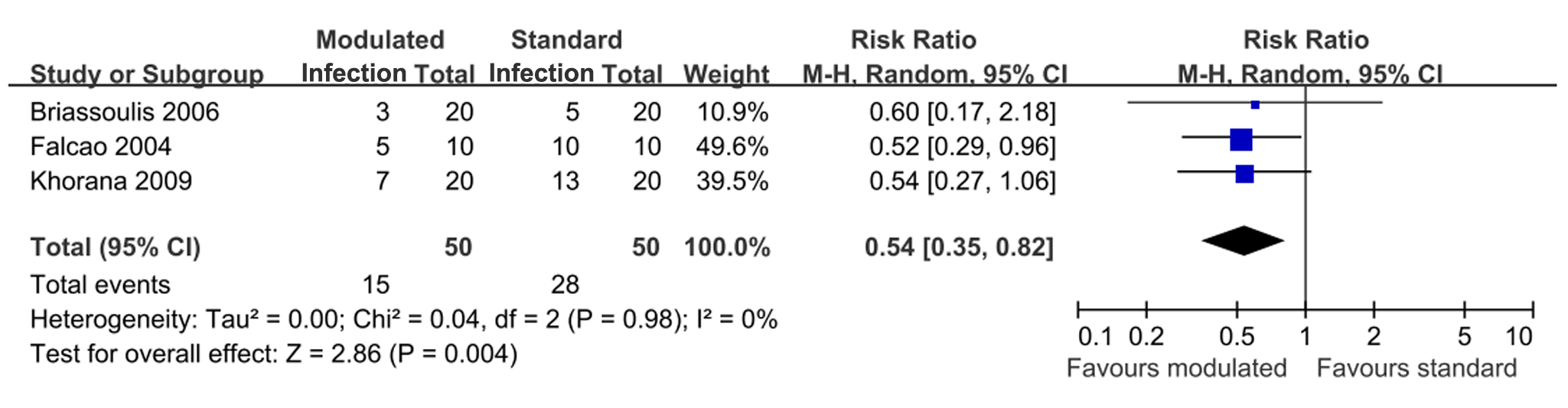

Supplement: Figure S3 — Forest plot shows the effect of standard and immuno-modulated nutritional formulae on infectious complications. (TIF) [file pone.0058838.s003.tif]

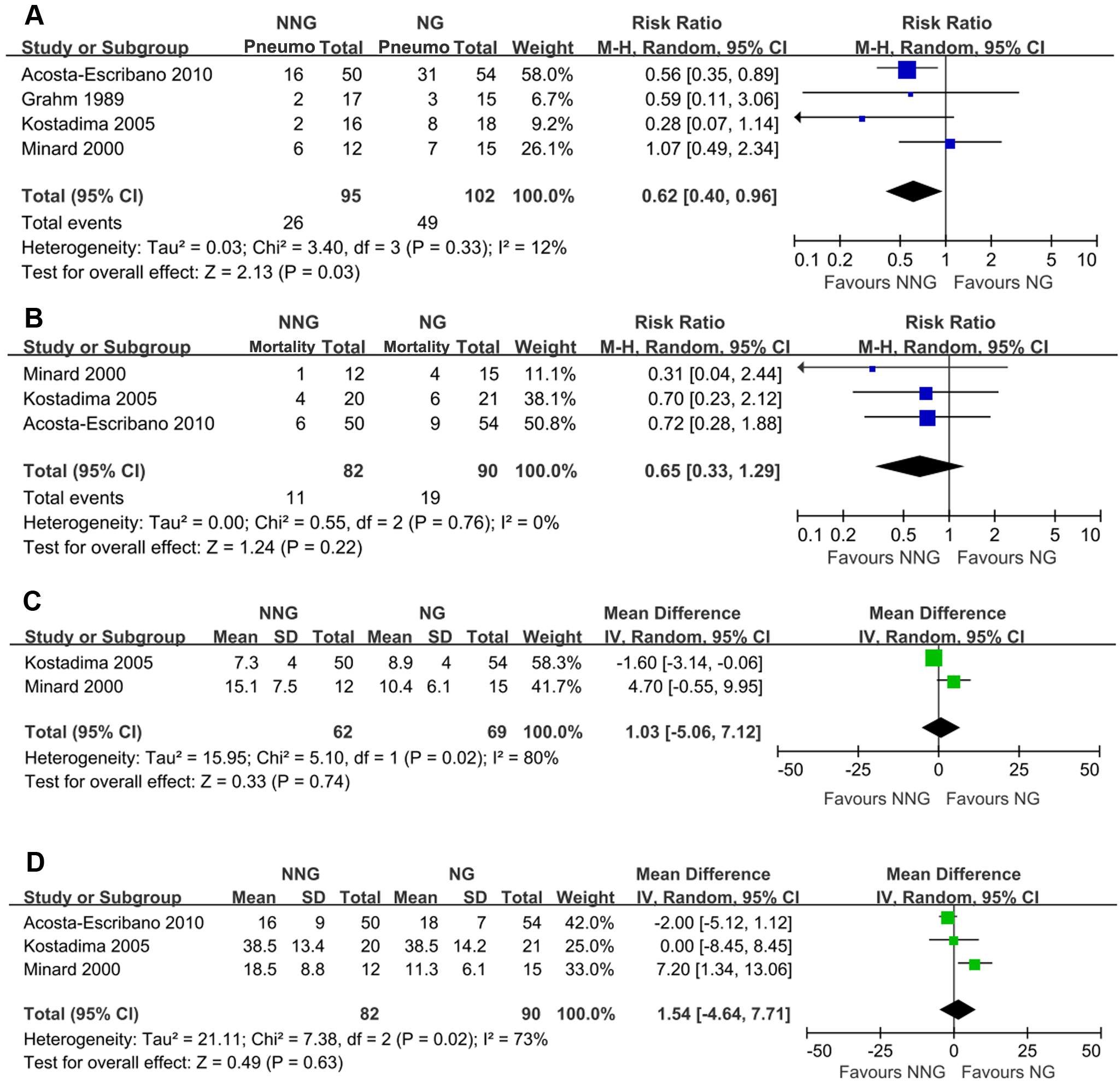

Supplement: Figure S4 — Forest plot shows the effect of non-nasogastric and nasogastric enteral feeding on outcomes in patients with TBI. (A) Forest plot illustrates the effect on pneumonia. (B) Forest plot shows the effect on mortality. (C) Forest plot shows the effect on ventilator days. (D) Forest plot shows the effect on length of stay in the intensive care unit. Pneumo, pneumonia. (TIF) [file pone.0058838.s004.tif]
