# Supplementary material for: Nutritional Support for Patients Sustaining Traumatic Brain Injury: A Systematic Review and Meta-Analysis of Prospective Studies
Source: PLoS One. 2013 Mar 19;8(3):e58838. doi: 10.1371/journal.pone.0058838 (PMC3602547; doi:10.1371/journal.pone.0058838)
Supplement: Protocol S1 — PRISMA Flowchart. (DOC) [file pone.0058838.s006.doc]

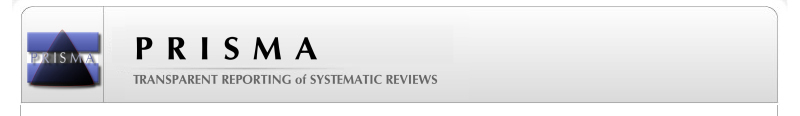
PRISMA 2009 Flow Diagram

**Screening**

**Included**

**Eligibility**

**Identification**

Records identified through database searching:
Ovid Medline (296)

Embase (384)

Cochrane Library (29)

(n = 709)

Records after duplicates removed
(n = 616)

Records screened
(n = 119)

Records excluded (n = 497)

Full-text articles assessed for eligibility
(n = 34)

Studies included in qualitative synthesis
(n = 24)

Studies included in quantitative synthesis (meta-analysis)
(n = 16)

8 without relevant outcomes

10 full-text articles excluded:

-5 not English

-1 compared combined EN/PN with TPN

-1 compared two fat emulsions

-1 compared essential amino acid with placebo

-1 compared intermittent with continuous EN

-1 compared different infusion speeds of EN
